# Musculoskeletal patients’ preferences for care from physiotherapists or support workers: a discrete choice experiment

**DOI:** 10.1186/s12913-024-11585-w

**Published:** 2024-09-19

**Authors:** Panos Sarigiovannis, Luis Enrique Loría-Rebolledo, Nadine E. Foster, Sue Jowett, Benjamin Saunders

**Affiliations:** 1 Primary Care Centre Versus Arthritis, School of Medicine, Keele University, Staffordshire, ST5 5BG UK; 2 Midlands Partnership University NHS Foundation Trust, Newcastle under Lyme, Staffordshire, ST5 2BQ UK; 3 Health Economics Research Unit, University of Aberdeen, Aberdeen, AB25 2ZD UK; 4 STARS Education and Research Alliance, Surgical Treatment and Rehabilitation Service (STARS), The University of Queensland and Metro North Health, , Herston, Brisbane, Australia; 5 Health Economics Unit, Institute of Applied Health Research, Public Health Building, University of Birmingham, Edgbaston, Birmingham, B15 2TT UK

**Keywords:** Musculoskeletal physiotherapy, Delegation, Support workers, Skill mix, Patient preferences, Discrete choice experiment, Continuity of care, Travel distance, Parking

## Abstract

**Background:**

Delegation of clinical tasks from physiotherapists to physiotherapy support workers is common yet varies considerably in musculoskeletal outpatient physiotherapy services, leading to variation in patient care. This study aimed to explore patients’ preferences and estimate specific trade-offs patients are willing to make in treatment choices when treated in musculoskeletal outpatient physiotherapy services.

**Methods:**

A discrete choice experiment was conducted using an efficient design with 16 choice scenarios, divided into two blocks. Adult patients with musculoskeletal conditions recruited from a physiotherapy service completed a cross-sectional, online questionnaire. Choice data analyses were conducted using a multinomial logit model. The marginal rate of substitution for waiting time to first follow-up physiotherapy appointment and distance from the physiotherapy clinic was calculated and a probability model was built to estimate the probability of choosing between two distinct physiotherapy service options under different scenarios.

**Results:**

382 patient questionnaires were completed; 302 participants were treated by physiotherapists and 80 by physiotherapists and support workers. There was a significant preference to be seen by a physiotherapist, have more follow-up treatments, to wait less time for the first follow-up appointment, to be seen one-to-one, to see the same clinician, to travel a shorter distance to get to the clinic and to go to clinics with ample parking. Participants treated by support workers did not have a significant preference to be seen by a physiotherapist and it was more likely that they would choose to be seen by a support worker for clinic scenarios where the characteristics of the physiotherapy service were as good or better.

**Conclusions:**

Findings highlight that patients treated by support workers are likely to choose to be treated by support workers again if the other service characteristics are as good or better compared to a service where treatment is provided only by physiotherapists. Findings have implications for the design of physiotherapy services to enhance patient experience when patients are treated by support workers. The findings will contribute to the development of “best practice” recommendations to guide physiotherapists in delegating clinical work to physiotherapy support workers for patients with musculoskeletal conditions.

**Supplementary Information:**

The online version contains supplementary material available at 10.1186/s12913-024-11585-w.

## Background

Musculoskeletal (MSK) conditions such as arthritis and low back pain are the leading cause of years lived with disability worldwide; they affect an estimated 20.2 million people across the UK, where they are the second leading cause of sickness absence from work [41]. Most MSK conditions can be managed in primary care or outpatient services in hospitals; evaluation and treatment by physiotherapists are frequently part of the treatment pathway [4]. Patients are assessed by physiotherapists and if they need follow-up treatments, these are provided by either a physiotherapist or a physiotherapy support worker.

The physiotherapy support worker role was developed to address some of the challenges affecting healthcare service delivery and the physiotherapy workforce worldwide. This includes an increasingly ageing population and an associated burden of healthcare; spiralling costs; increased patient expectations and a shortage of registered physiotherapists [3, 11] (Lizarondo et al., 2010; Munn et al., 2013). Physiotherapy support workers, who may also be known as physiotherapy assistants, rehabilitation assistants, technical instructors or physiotherapy technicians, are non-registered staff who work alongside physiotherapists to provide delegated interventions and responsibilities [33]. They do not hold a qualification accredited by a professional association and are not formally regulated by a statutory body.

In the UK, physiotherapy support workers form approximately 15% of the total physiotherapy workforce [37] and a significant proportion of them work in MSK physiotherapy services within the National Health Service (NHS). They may undertake any activity that is in pursuit of physiotherapy goals provided that the activity is delegated to them by a registered healthcare professional with appropriate supervision in place and, where necessary or indicated, access to support and advice from a registered physiotherapist [38]. However, physiotherapy support workers’ roles are relatively undefined and as such, there is considerable variation in the duties and tasks they undertake [31]. National guidance from the Chartered Society of Physiotherapy (CSP^1^) about delegation of tasks to support workers largely leave decision-making to the individual physiotherapist, their judgement of the task and their assessment of the competence of the support worker [39]. Consequently, in some physiotherapy services, physiotherapy support workers have a predominantly clinical role whereas in others they fulfil primarily an administrative role such as inputting data and booking appointments which leads to variation in clinical care [31].

A recent survey that explored current practice of UK MSK physiotherapists in relation to delegating clinical tasks to physiotherapy support workers, demonstrated that there is considerable variation in practice and delegation appears very patient-dependent [33]. This evidence suggests the need for a best practice framework to guide physiotherapists when delegating clinical tasks to support workers and standardise delegation. This need is being addressed through the Musculoskeletal Outpatient Physiotherapy Delegation (MOPeD) mixed methods study [34]. The first stage of the MOPeD study was a focused ethnographic study which explored how the culture in physiotherapy services may influence how tasks are delegated to physiotherapy support workers. It included detailed observations in two NHS musculoskeletal outpatient physiotherapy services and interviews with patients and clinicians. Stage 2a was a consensus study to reach agreement about what ‘best practice’ delegation recommendations should include.

A crucial step in the process of developing the ‘best practice’ recommendations is to understand musculoskeletal patients’ preferences about whether they are treated by physiotherapists or by physiotherapy support workers. The aim of the current study, which forms stage 2b of the MOPeD study, was to explore patients’ preferences about their care in MSK outpatient physiotherapy services and estimate specific trade-offs patients are willing to make in treatment choices when they are treated by physiotherapy support workers in MSK outpatient physiotherapy services.

## Methods

### Discrete choice experiment

A discrete choice experiment (DCE) was carried out. The DCE was conducted to elicit patients’ preferences when treated by physiotherapists and physiotherapy support workers in NHS MSK physiotherapy services. DCEs are an attribute-based survey method for measuring benefits (utility). Within healthcare, DCEs have been applied to address a wide range of issues in the delivery of healthcare including measuring and valuing attributes of a healthcare service and identifying the factors that influence choices and decisions of patients, the public and healthcare professionals [28, 35]. DCEs are based on the assumption that a service can be described by its characteristics or attributes, and the extent to which an individual values the service depends on the levels of these characteristics [36]. In a DCE, respondents are asked to choose between two or more alternatives, implicitly trading between the characteristics’ levels.

Ethical approval for the study was granted by the South West – Frenchay NHS Research Ethics Committee (REC) and the UK Health Research Authority (REC reference 21/SW/0158, IRAS project 297095).

### Attributes and levels

Development of the attributes and levels was guided by the qualitative findings from the first stage of the MOPeD study, which included semi-structured interviews of 19 patients who were treated by physiotherapists and support workers for a MSK condition (Sarigiovannis et al., in preparation), and further input from the study’s patient and public involvement and engagement (PPIE) and clinical advisory groups [34]. From the thematic analysis of the interviews, we identified factors that influenced patient preference. In addition, we explicitly asked participants of the PPIE group to identify the most important physiotherapy service characteristics that would influence their choice if they had to choose between two different physiotherapy services. The qualitative findings and the feedback from the PPIE and clinical advisory group were reviewed and combined to inform the selection of attributes and levels for the DCE [7]. The findings were reviewed by the PPIE group again and finally, seven attributes were included in the DCE. Table 1 shows the attributes and levels used in the MOPeD DCE.

**Table 1:** MOPeD DCE attributes and levels

Attributes	Description	Levels (coding)
Treating clinician	Who is treating you in your follow up sessions e.g., physiotherapists or physiotherapy assistants/support workers	- **Physiotherapists** (1) - Physiotherapy **assistants/support workers** (2)
Waiting times	How long you have to wait to be seen for your first follow-up session after your initial physiotherapy assessment	**− 2** weeks (1) **− 4** weeks (2) **− 6** weeks (3) **− 8** weeks (4)
Continuity of care	Treating clinician in your follow-up sessions i.e. if you are seen by the same or different person	Seen by the **same** person (1) Seen by **different** person (2)
Number of follow up treatments	If you have follow-up treatments and if you do, how many	**− 2** follow up (1) **− 4** follow ups (2) **− 6** follow ups (3) **− 8** follow ups (4)
Mode of follow up treatment	How you have your follow-up treatments e.g. one-to-one or in a group	**- One-to-one** with you and the therapist –**no** exercise equipment (1) - In an **exercise class** (gym) with other patients (2) **- One-to-one** with you and the therapist - **with** exercise equipment **(gym)** (3)
Distance to the clinic	How far you have to travel to get to the clinic	**− 2** miles (1) **− 4** miles (2) **− 8** miles (3) **− 16** miles (4)
Parking facilities	Availability of parking when attending your physiotherapy appointments	**- Limited** parking (1) **- Ample** parking (2)

### DCE design

For each choice task, participants were asked to select one of the two physiotherapy services (for further details refer to DCE instrument section). An opt out option was considered but it was decided not to include one as the choice frame reflects the real choice a patient would face. Additionally, it has been shown that inclusion of an opt out option results in small differences between the forced and unforced choice model while at the same time it compromises statistical significance [5, 40]. Given the attributes and levels, there were 1536 possible combinations of choice tasks (2^3^ × 3 × 4^3^). A D-efficient design, for a multinomial logit model (MNL)^2^ was used to select 16 choice tasks using the NGENE software. The design included an alternative specific constant (ASC) for the second alternative, which captures the likelihood of choice with respect to the first alternative when all attributes are held equal. The design was blocked into two versions of eight choice tasks [15, 21]. The final design was selected based on the lowest D-error, maximum level balance and utility balance, and minimal within-alternative correlation, minimal overlap [26, 29, 36].

### DCE instrument

**Fig. 1:**
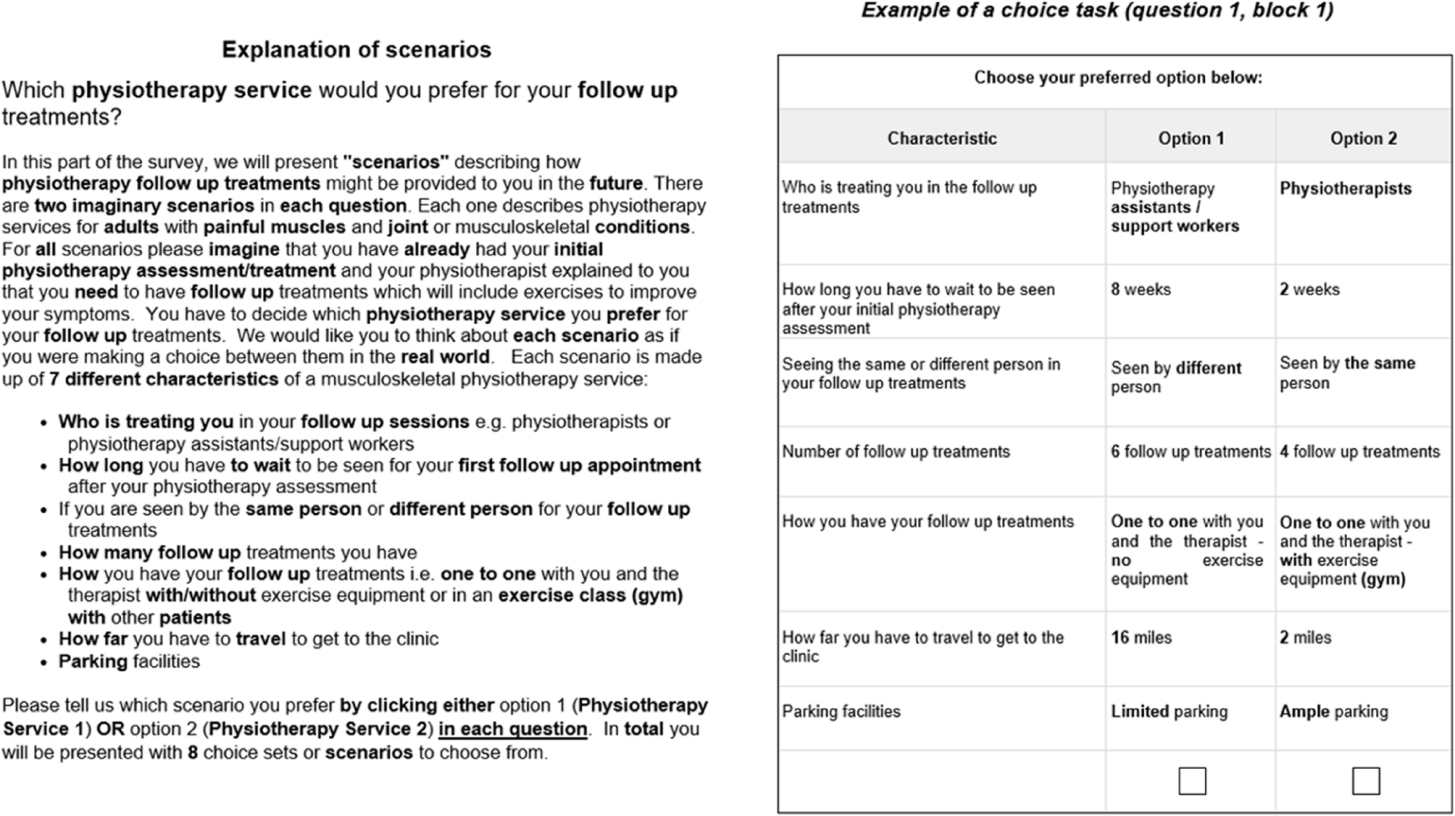
Explanation of scenarios and example of a choice task (question 1, block 1)

The DCE was administered as a cross-sectional questionnaire online survey with different sections which included questions about the participants’ health as well as sociodemographic data such as ethnicity, education and employment. The questionnaire was available online on the Qualtrics platform and participants completed it using tablet devices which were available at the participating clinics. However, a paper version of the questionnaire was produced to facilitate the ethical approval process (additional file 1). Participants were randomised to complete one of the two versions of the choice-set questions. An example of a choice-set question is shown in Fig. 1 together with the text that introduced the choice tasks.

The order of the choice tasks was randomised in each version. The questionnaire was piloted three times prior to the final version, to ensure ease of comprehension and test completion times. Following the first pilot, the order and wording of questions changed and after the second pilot an additional section about participants’ current treatment was added to facilitate comprehension. No changes were made after the third and final pilot. Depending on their answers, each participant was required to answer 22 to 25 questions in total. For example, only participants who answered that they used the car park at their physiotherapy clinic were asked to answer if the car park was ample or limited.

### Patient and public involvement and engagement

Meetings were held throughout the study with a group of seven patients to develop the DCE: three male and four female. All patients in the group had experience of treatment by a physiotherapist and/or physiotherapy support worker for a MSK condition. This support and feedback were essential in selecting the DCE attributes, designing and piloting the questionnaire, producing the participant.

information leaflets and interpreting the results. For example, based on the feedback from the PPIE group, it was decided to only offer the questionnaire in an online format which participants would complete using tablet devices in the participating physiotherapy clinics.

### Clinical advisory group

The design and methods of the DCE were also informed by discussion with a group of clinicians consisting of four physiotherapists, three physiotherapy support workers, one physiotherapy operational lead and one clinical lead. Based on the feedback received from the study’s clinical advisory group, it was decided that patient recruitment would be completed by the lead author, who was employed by the participating Trust, and three research facilitators. The group supported the delivery of the study, interpretation of results and dissemination.

### DCE sample size

The minimum sample size needed for DCEs depends on the specific hypotheses to be tested [10]. The sample size calculation was based on Orme’s formula which takes into account the number of choice tasks, the number of alternatives and the maximum number of levels for all attributes [24]. The absolute minimum number of participants required for the DCE would be 125 participants per block or 250 participants in total. However, as the need to conduct subgroup analysis was taken into consideration, it was decided to recruit between 180 and 200 per block or 360–400 participants in total [25].

### Participant eligibility criteria and recruitment

Adult patients (18 years old or older) who were treated for a MSK condition in one of the eight participating physiotherapy clinics in Midlands Partnership University NHS Foundation Trust (MPFT) were eligible to participate. The clinics were part of a primary care musculoskeletal physiotherapy service which served urban and rural areas covering the geographical area of North and South Staffordshire. Patients were referred to the physiotherapy service predominantly by general practitioners (GPs), first contact practitioners (FCPs) or advanced physiotherapy practitioners (APPs).

Physiotherapists within participating site(s), invited patients to self-complete the questionnaire when patients were either attending for their follow-up physiotherapy appointment or were discharged from treatment during pre-arranged recruitment days during the 4-month-recruitment period. Patients were directed to the researcher (PS) or the research facilitator when they expressed their interest to participate or to find out more information about the study. Patients who agreed to participate were asked to provide informed, written consent prior to completing the DCE questionnaire. Patients were provided with a tablet to complete the survey in a quiet office where the researcher (PS) or a research facilitator were available to help them if they encountered any difficulties with the equipment.

### Data analyses

Data analyses were conducted in STATA (Stata Corporation 2023, Stata Statistical Software: Release 18. College Station, Texas: Stata Corporation LLC). Data were analysed using an MNL model with robust standard errors. Additionally, subgroup analyses were conducted to identify the potential effect of observed patient characteristics [8]. For example, the sample was split into various subgroups including patients who were referred for physiotherapy because of a spinal condition or conditions other than spinal, patients with reduced or not reduced activities of daily living (ADL) due to their musculoskeletal condition(s), patients with or without at least a degree qualification, patients aged either under 65 years old or 65 years old and older, patients who were female or not and patients being treated by physiotherapists or by support workers. Marginal rates of substitution (MRS) (e.g., the ratio of two parameters) were calculated in terms of waiting times and travelling distance to allow direct comparison across subgroups which facilitated interpretation of data and account for observed heterogeneity [9, 12, 42]. Waiting times and travelling distance were selected since they have been reported to be important for patients [17] (Pitkänen and Linnosmaa, 2021). Finally, mean estimates were used to estimate the probability of choosing one of the two physiotherapy services based on scenarios of interest (e.g., between services provided by physiotherapist and support worker).

## Results

### Study participants

382 patients were recruited, with 232 (60.73%) attending their physiotherapy appointments within North Staffordshire and 150 (39.27%) in South Staffordshire. Mean completion time of the online survey was 11.16 min. 251 participants (65.71%) were female and 190 patients (49.74%) were 65 years old or older. 371 participating patients (97.12%) described their ethnic group as “white”. 155 participants (40.57%) reported having no qualifications, whereas 142 (37.17%) held at least a degree qualification. 192 participants (50.26%) were retired and 117 (30.63%) preferred not to reveal their household income. 176 (46.07%) participants stated that they suffered from long-term conditions which affected their activities of daily living. Characteristics of the study participants are shown in Table 2.

**Table 2:** Participants’ characteristics

	Number of respondents / (382)	Percentage of respondents
**Gender**		
Male	131	34.29%
Female	251	65.71%
**Age group**		
18–44	51	13.35%^a^
45–64	141	36.90%
65+	190	49.74%
**Ethnicity**		
White	371	97.12%
Black	3	0.78%
Asian	4	1.05%
Mixed	4	1.05%
**Education**		
No qualifications	155	40.57%^a^
Entry level (including A level)	85	22.25%
Higher education	142	37.17%
**Employment status**		
Working as an employee	121	31.67%^a^
Self-employed or freelance	23	6.02%
Temporarily away from work ill, on holiday or laid off	14	3.66%
Retired	192	50.26%
Looking after home or family	5	1.31%
Long term sick or disabled	17	4.45%
Other	10	2.62%
**Annual household income**		
Less than £20,000	109	28.53%^a^
£20,000-£39,000	89	23.30%
£40,000-£59,000	42	10.99%
£60,000-£99,000	20	5.23%
More than £100,000	5	1.31%
Prefer not to say	117	30.63%
**Geographical area of the clinic treated**		
North Staffordshire	232	60.73%
South Staffordshire	150	39.27%
**Reason for MSK physiotherapy referral**		
Spinal symptoms	125	32.72%
No spinal symptoms	257	67.28%
**History of long-term conditions affecting activities of daily living**		
N/A (No long-term conditions)	184	48.17%
Yes	176	46.07%
No	22	5.76%

^a^Only two decimal points are displayed in the figures and due to rounding, percentages do not add up to 100%

### MNL model analysis

There was no evidence the position of the alternative scenario impacted the likelihood of choosing a service (e.g., no left to right bias). The results (Table 3) showed patients prefer being seen by a physiotherapist as opposed to a support worker; waiting less time to be seen for the first follow up appointment and receiving continuity of care. Furthermore, the results indicated that patients prefer to have more follow-up treatments; travel less distance to get to the physiotherapy clinic and have appointments in clinics with ample parking. Finally, being treated in an exercise class with other patients was preferred less than when being treated one-to-one without equipment. However, when the treatment was delivered one-to-one, they were indifferent to having access to equipment. Appendix shows the MNL model analysis for the relevant subgroups.

**Table 3:** MOPeD DCE multinomial logit model of patient preferences (std. Err. Adjusted for 382 clusters in id)

**Variables and reference levels**			**Coef.**	**Std error**	***p*-value**	**95% Conf Interval**
*Alternative Specific Constant (asc)*						
ref: asc1						
*** Asc 2***			0.020	0.0351045	0.551	[-0.047, 0.089]
*Treating clinicians*						
ref: physiotherapist						
*** Physiotherapy assistant/support worker***			-0.445	0.0550134	**<0.001**	[-0.553, -0.338]
*Waiting time for FU treatments*						
*** Wait: number of weeks (2-8)***			-0.061	0.0104963	**<0.001**	[-0.82, -0.41]
*Continuity of treatment*						
ref: same clinician						
*** Different clinician***			-0.787	0.0575207	**<0.001**	[-0.9, -0.675]
*Number of follow up treatments*						
*** FU: number follow up treatments (2-8)***			0.030	0.0091500	**0.001**	[0.012, 0.048]
*Mode of treatment*						
* ref: mode 1 (One-to-one without equipment)*						
*** Mode 2: In an exercise class (gym) with other patients***			-0.466	0.0667437	**<0.001**	[-0.598, -0.336]
*** Mode 3:One-to-one with you and the therapist - with exercise equipment (gym)***			0.022	0.0609921	0.713	[ -0.97, 0.142]
*Distance to the clinic*						
*** Distance: number of miles (2-16)***			-0.075	0.0055528	**<0.001**	[-0.87, -0.65]
*Parking at the clinic*						
* ref: limited parking*						
*** Ample parking***			0.230	**.0391029**	**<0.001**	[0.154, 0.307]
*Number of total choices: 6112* *Log pseudolikelihood: -1716.461*		*Akaike crit. (AIC): 3450.922* *Bayesian crit. (BIC): 3511.384*				

### Marginal rates of substitution for waiting time and distance

**Fig. 2:**
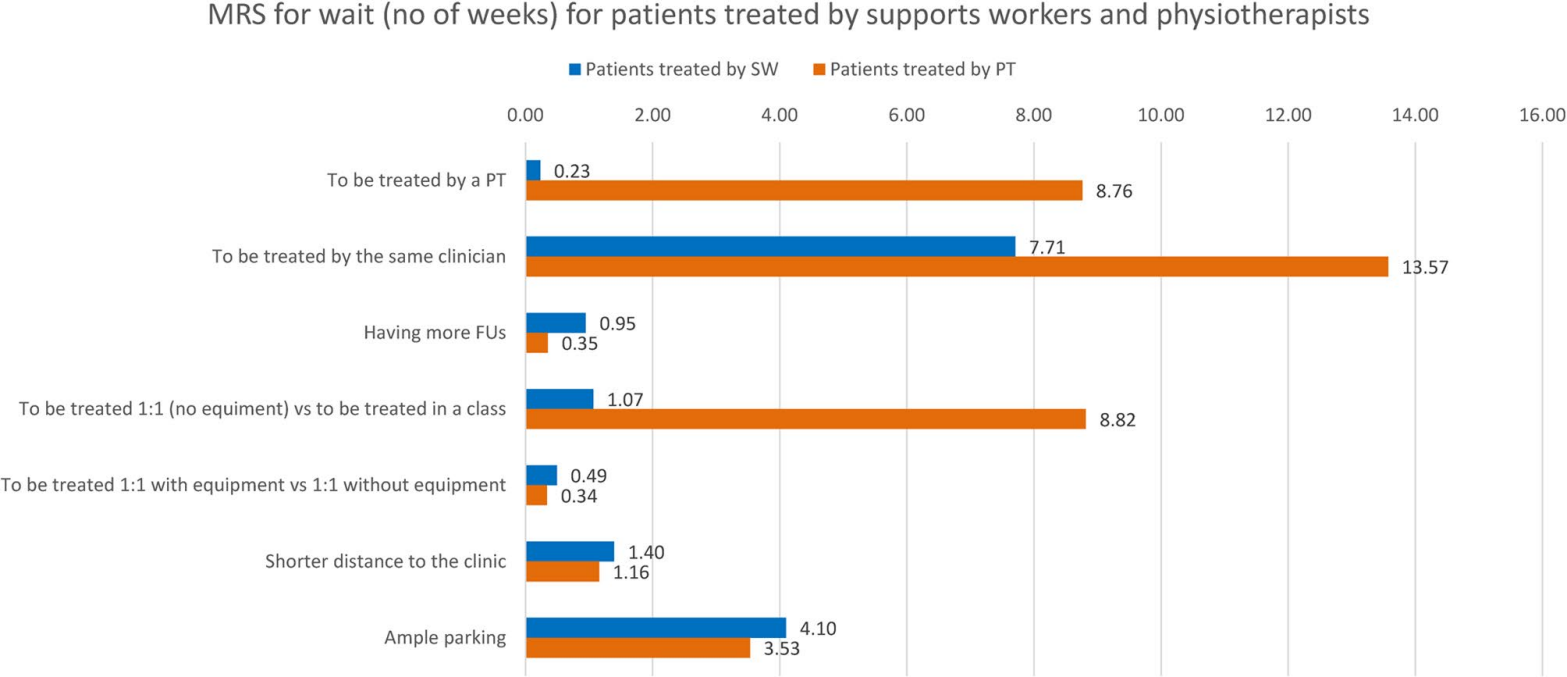
Willingness to wait values to get a marginal change per attribute for patients treated by support workers and physiotherapists

Figure 2 shows the MRS in terms of waiting time for the two subgroups: patients currently treated by support workers and those treated by physiotherapists. Patients treated by physiotherapists were willing to wait an additional period of 8.76 weeks to be seen by a physiotherapist, 13.57 weeks to be treated by the same clinician for their follow-up treatments, 0.35 weeks for each additional follow-up appointment, 8.82 weeks to be treated one-to-one without equipment instead for being treated in a class with other patients, 0.34 weeks to be seen one-to-one with equipment instead of one-to-one without equipment, 1.16 weeks per mile being closer to the physiotherapy clinic and 3.53 weeks to go to a clinic with an ample parking. Patients who were treated by physiotherapy support workers were willing to wait an additional period of 0.23 weeks to be seen by a physiotherapist, 7.71 weeks to be treated by the same clinician for their follow-up treatments, 0.95 weeks for each additional follow-up appointment, 1.07 weeks to be treated one-to-one without equipment, 0.49 weeks to be seen one-to-one with equipment, 1.40 weeks per mile being closer to the physiotherapy clinic and 4.10 weeks to go to a clinic with an ample parking.

**Fig. 3:**
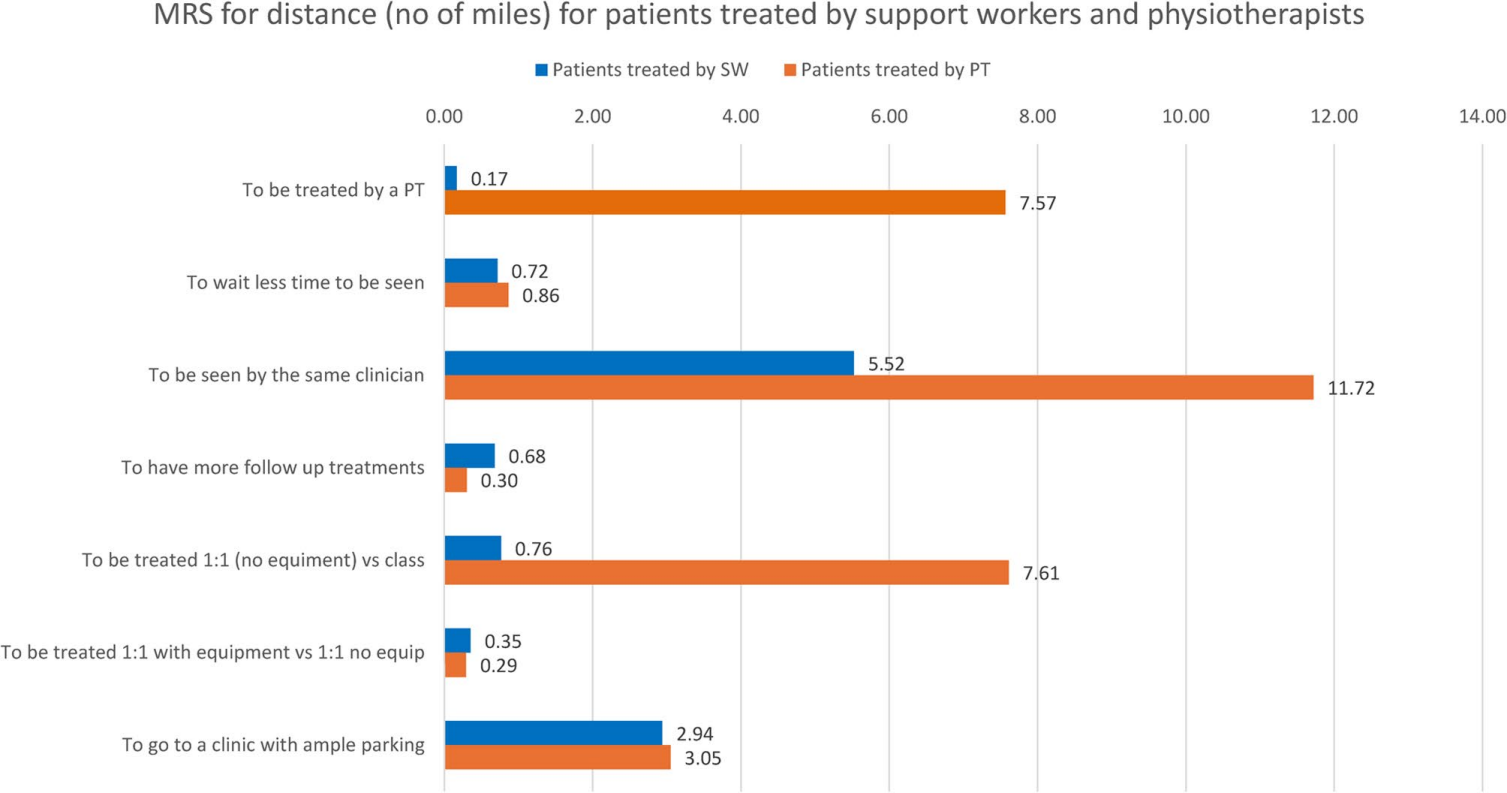
Willingness to travel values to get a marginal change per attribute for patients treated by support workers and physiotherapists

Figure 3 shows the MRS for travelling distance for the same subgroups. Patients treated by physiotherapists were willing to travel an additional distance of 7.57 miles to be seen by a physiotherapist, 0.86 miles for each week that they have to wait less to be seen for their first follow-up appointment, 11.72 miles to be treated by the same clinician for their follow-up treatments, 0.30 miles for each additional follow-up appointment, 7.61 weeks to be treated one-to-one without equipment instead for being treated in a class with other patients, 0.29 miles to be seen one-to-one with equipment instead of being treated one-to-one without equipment, and 3.53 miles to go to a clinic with an ample parking. Patients treated by physiotherapy support workers were willing to travel an additional distance of 0.17 miles to be seen by a physiotherapist, 0.72 miles for each week that they have to wait less to be seen for their first follow-up appointment, 5.52 miles to be treated by the same clinician, 0.68 miles for each additional follow-up appointment, 0.76 miles to be treated one-to-one without equipment instead for being treated in a class, 0.35 miles to be seen one-to-one with equipment, and 2.94 miles to go to a clinic with an ample parking.

### Probability of choosing services

Table 4 shows the probability of participants selecting one of the two physiotherapy service options listed in three different scenarios with two services each. Scenario 1 describes a choice between a service (*Service 1*) where patients are treated by physiotherapists and one (*Service 2*) where patients are treated by physiotherapy support workers in which other service characteristics are seemingly less favourable (e.g., farther away and more waiting time). Scenario 2 is similar to Scenario 1 with the only difference being the distance to the physiotherapy clinic has been reversed between the two services (i.e. 16 miles distance for *Service 1* and 2 miles distance for *Service 2*). Scenario 3 also describes a *Service 1* where the treatment is provided by physiotherapists and a *Service 2* where treatment is provided by support workers, but with other service characteristics that seemingly favour the latter.

**Table 4:** Probability to select a MSK physiotherapy service in three scenarios for separate groups of participants

	Scenario 1		Scenario 2		Scenario3	
Service characteristics	Service 1	Service 2	Service 1	Service 2	Service 1	Service 2
Treating clinician	Physiotherapist	Support worker	Physiotherapist	Support worker	Physiotherapist	Support worker
Distance to the clinic (miles)	2 miles	16 miles	16 miles	2 miles	8 miles	4 miles
Waiting time for first follow up appointment (weeks)	2 weeks	8 weeks	2 weeks	8 weeks	4 weeks	2 weeks
Number of follow up appointments	6 follow up appointments	6 follow up appointments	6 follow up appointments	6 follow up appointments	2 follow up appointments	6 follow up appointments
Mode of treatment	One-to-one with equipment	In a class with other patients	One-to-one with equipment	In a class with other patients	One-to-one without equipment	In a class with other patients
Seeing the same or different clinician	Same	Different	Same	Different	Same	Same
Parking at the clinic	Ample	Limited	Ample	Limited	Limited	Ample
**Participants/subgroups**	**Probability to choose one of the two services in each scenario**					
All participants (*N* = 382)	96.72%	3.28%	77.88%	22.12%	53.34%	46.66%
Participants treated by physiotherapists (*N* = 302)	97.80%	2.20%	83.81%	16.19%	59.82%	40.18%
Participants treated by support workers (*N* = 80)	90.74%	9.26%	50.10%	49.90%	30.44%	69.56%

Results showed that in Scenario 1, on average most patients (96.72%) would choose the service with the physiotherapist (*Service 1*). Furthermore, who the patient’s current clinician is does not seem to impact the likelihood of choice: 97.80% patients being treated by physiotherapists and 90.74% patients being treated by support workers would choose the service with the physiotherapist (*Service 1*). Similarly, in Scenario 2, on average most patients (77.88%) would choose the service with the physiotherapist (*Service 1)*. However, given the change in travel distances to each clinic, there was a bigger difference between the two subgroups: 83.81% of patients treated by physiotherapists would choose *Service 1* compared to 50.10% of patients treated by support workers.

In Scenario 3, given the least favourable service characteristics of the service where the treatment is provided by physiotherapists (*Service 1*), on average the choice proportions between the two services were more equally split with 46.66% of patients would choose a service with a support worker. At the same time, there was also evidence that current treating clinician had an effect on probabilities: 69.56% of patients treated by support workers were likely to choose the service which included a support worker versus 40.18% of those treated by physiotherapists. Conversely, 59.82% of participants who have been seen by a physiotherapist versus 30.44% of those seen by a support worker would choose a service where treatment is provided exclusively by physiotherapists even when the other characteristics are less favourable.

## Discussion

### Main findings

This paper reports the findings of a study which used DCE methodology to elicit patients’ preferences when they are treated by physiotherapists and physiotherapy support workers in MSK outpatient physiotherapy services. Patients elicited expected preferences. Nevertheless, the results of this DCE showed that patients’ experience of being treated by a physiotherapist or support worker previously is a significant factor when making a choice about the future. Patients who had been seen by support workers were likely to choose to be seen by a support worker again provided the other service characteristics such as travelling distance, parking facilities or number of follow-up treatments, are similar or marginally better when compared to those of a physiotherapy service where follow up treatments are provided by physiotherapists.

### Comparison with previous research literature

Charles et al. [6] conducted a DCE to explore older patients’ preferences for hip fracture rehabilitation services. The DCE attributes included whether the treating clinician was a qualified physiotherapist/occupational therapist or a support worker. The authors reported that there was a significant preference for the healthcare professional delivering the rehabilitation sessions to be a qualified physiotherapist or occupational therapist. Nevertheless, their results should be viewed with caution as the study sample used in their DCE was small (41 patients). Additionally, Charles et al. [6] described support workers in their DCE as “supervised unqualified assistants” which may have been confusing and misleading for participants. Physiotherapy support workers are non-registered clinicians but not unqualified as the vast majority of them have qualifications that enable them to work safely and effectively in their role [31].

Uncertainty and lack of clarity in relation to support workers’ role as well as lack of previous experience of being treated by a physiotherapy support worker, may have been among the factors which influenced patients, who were not treated by support workers in our study, to choose to be treated by a physiotherapist instead of a support worker. Historically, there has been a lack of regulation and registration for physiotherapy support workers, and as a result, many of these positions have evolved with variations in the title, and an inconsistent understanding of the role as well as the educational and supervision requirements [14, 32].

Convenience is a well-known factor for treatment choice. It has been reported that patients tend to choose their preferred location taking into account parking availability, duration of trip and transport services’ availability and costs (Perry et al., 2015, Pitkänen and Linnosmaa 2021). Pitkänen and Linnosmaa (2021) reported that, in general, all patients prefer high-quality providers within short distances. This is supported by the findings of this study as patients preferred to travel the shortest possible travelling distance to get to the physiotherapy clinic. Nevertheless, patients in our study were prepared to travel longer distances to be seen by the same clinician. Continuity of care of care is known to have important benefits for patients, healthcare professionals and healthcare systems [13, 16, 22].

Mason [23] reported that between 70 and 90% of patients who accessed hospitals used cars to attend for outpatient appointments and that and the parking experience could be an additional source of financial pressure, worry and stress, which appeared to affect patient satisfaction principally when it was either very good or very bad. Participants in our sample clearly demonstrated their significant preference for physiotherapy clinics with ample parking.

Physiotherapy treatments for MSK conditions are usually delivered on a one-to-one basis or in group settings. There is evidence that physiotherapy treatments provided in groups are similarly effective to one to one care and cost-effective [1, 30]. While group-based treatments may be appropriate for some patients, they will not suit all patients [19]. Participants in our study preferred to be seen one to one instead of in a group; however, patients treated by support workers did not have a strong preference to be seen one to one. It is reported that healthcare providers increasingly seek to improve quality of patient care by focusing on patients’ needs and preferences, with ‘patient-centredness’ recognised as a domain of quality in its own right [27]. To implement patient-centred care, it is essential that clinicians take into consideration patient preferences when they formulate their treatment plan [18].

### Strengths and limitations

A robust process was followed in designing the DCE, involving incorporation of findings of the previous stages of the MOPeD study and input from different stakeholders, including patients. This process was reflected in the face validity of the analysis results. Additionally, a face-to-face data collection approach in this DCE resulted in high response rate and in receiving no incomplete questionnaires. Finally, we recruited 382 patients which allowed us to conduct various subgroup analyses. However, this study comes with limitations. First, the data analysis used a conditional or multinomial logit model (MNL) which has restrictive assumptions. For example, independence or irrelevant alternatives, independent and identically distributed errors and identical preferences across respondent e.g., no preference homogeneity [20, 29]. An MNL was used, instead of a more flexible model that explores unobserved heterogeneity (but also contains requires other assumptions), to focus on more policy actionable observed heterogeneity as shown in the subgroup analysis and calculation of MRS and probabilities.

Second, linearity of the continuous variables was not tested because these were designed as continuous variables and not as categorical. The reason for this design specification was to minimise the degrees of freedom needed to estimate the model given the expected sample size [2]. Furthermore, the decision to treat them as continuous variables was supported by the guidance from the literature and the clinical advisory group of the study given the focus and ease of interpretation of the results if presented as marginal rates of substitution.

Third, participation of the study was limited to patients attending appointments in the eight participating physiotherapy MSK clinics within MPFT in North and South Staffordshire. Although the participants sample was not a representative sample of the UK population of MSK patients for ethnicity as most participants described their ethnic group as “white”, it was representative of the population of MSK patients that attend physiotherapy appointments in North and South Staffordshire. Partial postcode data were collected (the first part of the participants’ postcode) to ensure anonymity and comply with ethical approval but it was not possible to link the collected postcode data to full postcode data from other studies and such comparisons could not be made. We thus acknowledge that our findings may not be generalisable to all UK MSK patients including those who attend physiotherapy appointments for a MSK condition in the private sector. Nevertheless, the findings of this study highlight issues which are not pertinent exclusively to the NHS or the MSK clinical setting in relation to patients’ preferences when they are treated by support workers. Therefore, the findings may have wider applicability beyond the MSK setting or the NHS context and as such may be useful for informing research and practice in other settings both in the UK and internationally.

## Conclusions and implications

The findings reported in this paper provide evidence about patients’ preferences as well as what service characteristics patients consider important when they are treated by physiotherapy support workers in MSK outpatient physiotherapy services. The findings demonstrated that patient experience of being treated by a physiotherapist or support worker is a significant factor when making a choice about the future. Unless patients have the experience of being treated by a physiotherapy support worker, they prefer to be treated by a physiotherapist regardless of any other less favourable characteristics of the physiotherapy service such as longer waits, distance and limited parking. Even when patients are treated by support workers, they seem to choose to be seen by a support worker again only when the other service characteristics are as good or favourable when compared to a service where treatment is provided only by physiotherapists.

The findings of this study indicate that there is a lack of understanding of the support workers’ role. They also suggest that it would be useful for patients to be given a clear explanation about the support worker role, their capabilities and why they are the most suitable and competent person for particular aspects of their treatment, in order to increase patients’ confidence and acceptability in being seen by a support worker. Finally, our findings highlight that continuity of care is important for patients. Within the study’s limitations, these findings can inform design of a physiotherapy service delivery to enhance patient experience when patients are treated by physiotherapists and support workers.

The findings of this study will be triangulated together with the results from the other stages of the MOPeD study to directly inform the development of “best practice” recommendations which incorporate the preference of patients, to guide physiotherapists to delegate clinical tasks to physiotherapy support workers in the MSK setting.

## Supplementary Information

Supplementary Material 1

## Data Availability

The data that support the findings of this study are not openly available due to reasons of sensitivity and are available from the corresponding author upon reasonable request.

## Appendix

**Table:** MNL model analysis - coefficient values for all participants and various subgroups^a^

Variables and levels	ALL	Spinal = 1	Spinal = 0	ADL = 1	ADL = 0	Degree = 1	Degree = 0	SW = 1	SW = 0	65+ = 1	65+ = 0	Female = 1	Female = 0
Alternative Specific Constant (asc)													
**Asc2**	0.02	0.07	-0.003	0.015	0.025	0.0383	0.01	-0.027	0.031	0.002	0.047	-0.013	0.086
Treating clinician													
**Support worker**	-0.445	**-0.559**	**-0.398**	**-0.486**	**-0.411**	**-0.554**	**-0.383**	-0.013	**-0.58**	**-0.360**	**-0.533**	**-0.412**	**-0.527**
Waiting time for FU treatments													
**Number of weeks**	-0.061	**-0.089**	**-0.049**	**-0.056**	**-0.064**	**-0.074**	**-0.054**	**-0.058**	**-0.066**	**-0.042**	**-0.082**	**-0.049**	**-0.084**
Continuity of treatment													
**Different clinician**	-0.787	**-0.943**	**-0.728**	**-0.711**	**-0.856**	**-0.847**	**-0.759**	**-0.449**	**-0.899**	**-0.706**	**-0.862**	**-0.802**	**-0.770**
Number of follow up (FU) treatments													
**Number of FUs**	0.030	0.027	**0.033**	**0.032**	**0.027**	**0.031**	**0.030**	**0.055**	**0.023**	**0.028**	**0.031**	**0.036**	0.021
Treatment mode													
**In a class**	-0.466	**-0.536**	**-0.441**	**-0.466**	**-0.463**	**-0.363**	**-0.532**	-0.062	**-0.584**	**-0.329**	**-0.602**	**-0.549**	**-0.311**
**One-to-one with equipment**	0.022	-0.014	0.038	-0.027	0.072	0.152	**-0.053**	0.028	0.022	0.053	0	0	0.079
Distance to the clinic													
**Number of miles**	-0.075	**-0.066**	**-0.081**	**-0.083**	**-0.069**	**-0.065**	**-0.082**	**-0.081**	**-0.076**	**-0.077**	**-0.074**	**-0.077**	**-0.072**
Parking at the clinic													
**Limited parking**	**0.230**	**0.266**	**0.217**	**0.174**	**0.277**	**0.226**	**0.238**	**0.239**	**0.234**	**0.207**	**0.255**	**0.202**	**0.283**

Note: Due to scaling issues, the magnitude of the coefficient cannot be directly used to compare preference estimates. However, the significance provides insight as to how preferences change across subgroups

^a^Coefficient values in bold indicate that the p value ≤ 0.05

## Abbreviations

ADL: Activities of Daily Living
APPs: Advanced Physiotherapy Practitioners
ASC: Alternative Specific Constant
CSP: Chartered Society of Physiotherapy
DCE: Discrete Choice Experiment
FCPs: First Contact Practitioners
GPs: General Practitioners
IRAS: Integrated Research Application System
MNL: Multinomial Logit
MOPeD: Musculoskeletal Outpatient Physiotherapy Delegation
MPFT: Midlands Partnership University NHS Foundation Trust
MRS: Marginal Rates of Substitution
MSK: Musculoskeletal
NHS: National Health Service
PPIE: Patient and Public Involvement and Engagement
REC: Research Ethics Committee
UK: United Kingdom

## Footnotes

1 The Chartered Society of Physiotherapy (CSP) is the professional, educational and trade union body for the UK’s chartered physiotherapists, physiotherapy students and support workers.

2 The MNL model is described as the ‘workhorse of choice modelling’ and it has been recommended as a natural first model to estimate [20].
