# Supplementary material for: Musculoskeletal patients’ preferences for care from physiotherapists or support workers: a discrete choice experiment
Source: BMC Health Serv Res. 2024 Sep 19;24:1095. doi: 10.1186/s12913-024-11585-w (PMC11411776; doi:10.1186/s12913-024-11585-w)

**Additional file 1: Paper version of the questionnaire used in the discrete choice experiment survey (MOPeD study) produced exclusively to facilitate the ethical approval process**

**Important note:** The DCE will be administered on-line using the link below:

**(TBC on submission)**

The content is reproduced here, but the on-screen format cannot be captured.

Participants will be randomised to complete **either** block 1 **or** block 2 of the choice-set questions. Questions in block 1 and block 2 will appear in random order.

**Introduction to the survey**


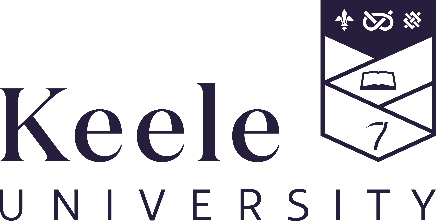

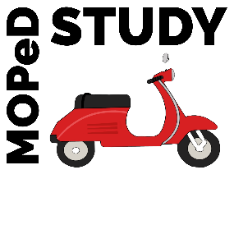

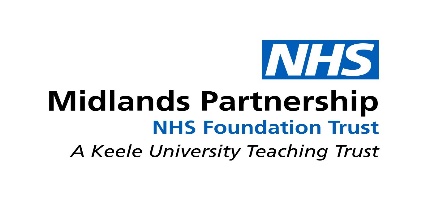


**M**usculoskeletal **O**utpatient **P**hysioth**e**rapy **D**elegation Survey

Dear **physiotherapy user**

You are being **invited** to take part in a **research study** by completing a **survey** on **physiotherapy practices** for **adults** with painful muscles and joint or **musculoskeletal conditions**. **Before** you **decide** whether to **take part**, it is important for you to **understand** **why** the research is **being done** and what it will **involve**. Please **read** the **survey participant information** sheet for additional details about the survey before completing it and take time to read the following information carefully**. Participation/no participation** in the survey **will not affect** your current physiotherapy treatment.


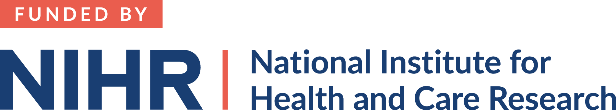


**Introduction to the survey**

Dear physiotherapy service user,
We are conducting this survey in **musculoskeletal physiotherapy departments across Staffordshire**to gain a better understanding about **patients' preferences in relation to their physiotherapy treatment**. As a guide, the people involved in piloting the survey said it took them approximately **15 minutes** to complete it.

At the **beginning of the survey**, you will be asked generic questions about you and **your health**. You will then be presented with **a series of scenarios that describe different physiotherapy services**, and we will ask you to **choose which one you would prefer**. The scenarios will differ in terms of **specific characteristics** of physiotherapy services, which allows us to explore **people's preferences** on different aspects of healthcare services.  At the **end of the survey**, you will be asked **questions about you** (e.g., where you live, qualifications, employment etc.). These will help us make sense of the results of the survey.

Please read the**survey participant information sheet** for **additional details** about the survey **before completing it**.  If you require any **further information**about this study, please contact Panos Sarigiovannis **via e-mail:** p.sarigiovannis1@keele.ac.uk
By completing and submitting the questionnaire, you agree that **any information you provide** can be used by **Keele University** for **research purposes only**. **No information** will be used that could lead to **you**being **identified personally**so your responses will be**anonymous.**

**Questions about you and your health**

The next set of questions are about you and your health

**QH1: How old are you? Please enter your AGE**


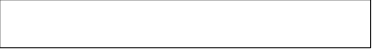


Skips To: End of Survey If Condition: How old are you? …..Is Less Than 18.

**QH2: How do you describe yourself?**

- Male
- Female
- Non-binary/ third gender
- Prefer to self describe


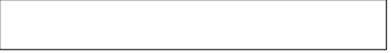


- Prefer not to say

**QH3: How is your health in general?**

- Very good
- Good
- Fair
- Bad
- Very bad

**QH4: Do you have any physical or mental health conditions or illnesses lasting or expected to last 12 months or more?**

- Yes (Go to question QH5)
- No (Go to question QH6)

**QH5: Do any of your conditions or illnesses reduce your ability to carry out day-to-day activities?**

- Yes
- No

**QH6: Please indicate the main body part/main reason you are having/had physiotherapy treatment for. For multiple areas please select all that apply**

- Upper limb
- Lower limb
- Back
- Neck

**QH7: Who is treating you/treated you during your physiotherapy treatments?**

- Physiotherapist only
- Physiotherapist and physiotherapy assistant/support worker
- Not sure / don’t know

**QH8: Please indicate how you usually get to the physiotherapy clinic(s). Select all that apply**

- On foot
- Cycling
- Driving own car
- Taxi
- Having a lift
- Public transport
- Other, please specify

**Explanation of "scenarios"**

**Explanation of "scenarios"**
In the next section you will be presented with **two imaginary "scenarios"**in**each question**. Each scenario describes physiotherapy services for adults with painful muscles and joint or musculoskeletal conditions.
For **all**scenarios please **imagine** that you have**already**had your**initial physiotherapy assessment/treatment**and your physiotherapist explained to you that you **need** to have **follow up**treatments which will include exercises to improve your symptoms.  You have to decide which **physiotherapy service** you **prefer** for your**follow up**treatments.  We would like you to think about**each scenario** as if you were making a choice between them in the**real world**.   Each scenario is made up of **7 different characteristics**of a musculoskeletal physiotherapy service:

- **Who is treating you** in your **follow up sessions** e.g. physiotherapists or physiotherapy assistants/support workers
- **How long**you have **to wait** to be seen for your **first follow up appointment** after your physiotherapy assessment
- If you are seen by the **same person**or**different person**for your **follow up** treatments
- **How many follow up**treatments you have
- **How**you have your **follow up**treatments i.e. **one to one** **with you and the therapist with/without** exercise equipment or in an **exercise class with other patients**
- **How far** you have to **travel** to get to the clinic
- **Parking** facilities

Please tell us which scenario you prefer **by clicking either**option 1 (**Physiotherapy Service 1**) **OR**option 2 (**Physiotherapy Service 2**) **in each question**.  In **total** you will be presented with **8**choice sets or **scenarios** to choose from.

**BLOCK 1**

**Scenario 1 (IA)**

**Choose your preferred option below:**

| **Characteristic** | **Option 1** | **Option 2** |
| --- | --- | --- |
| Who is treating you in the follow up treatments | **Physiotherapists** | Physiotherapy **assistants / support workers** |
| How long you have to wait to be seen after your initial physiotherapy assessment | **6** weeks | **4** weeks |
| Seeing the same or different person in your follow up treatments | Seen by **different** person | Seen by **the same** person |
| Number of follow up treatments | **6** follow up treatments | **4** follow up treatments |
| How you have your follow up treatments | **One to one** with you and the therapist - with exercise equipment **(gym)** | In an exercise **class (gym) with** other **patients** |
| How far you have to travel to get to the clinic | **16** miles | **2** miles |
| Parking facilities | **Ample** parking | **Limited** parking |
|  |  |  |

**Scenario 2 (IIA)**

Choose your preferred option below:

| **Characteristic** | **Option 1** | **Option 2** |
| --- | --- | --- |
| Who is treating you in the follow up treatments | **Physiotherapists** | Physiotherapy **assistants / support workers** |
| How long you have to wait to be seen after your initial physiotherapy assessment | **8** weeks | **2** weeks |
| Seeing the same or different person in your follow up treatments | Seen by **the same** person | Seen by **different** person |
| Number of follow up treatments | **4** follow up treatments | **6** follow up treatments |
| How you have your follow up treatments | In an exercise **class (gym) with** other **patients** | **One to one** with you and the therapist - **no** exercise equipment |
| How far you have to travel to get to the clinic | **16** miles | **2** miles |
| Parking facilities | **Limited parking** | **Ample** parking |
|  |  |  |

**Scenario 3 (IIIA)**

Choose your preferred option below:

| **Characteristic** | **Option 1** | **Option 2** |
| --- | --- | --- |
| Who is treating you in the follow up treatments | **Physiotherapists** | Physiotherapy **assistants / support workers** |
| How long you have to wait to be seen after your initial physiotherapy assessment | **2** weeks | **8** weeks |
| Seeing the same or different person in your follow up treatments | Seen by **the same** person | Seen by **different** person |
| Number of follow up treatments | **2** follow up treatments | **8** follow up treatments |
| How you have your follow up treatments | **One to one** with you and the therapist - **no** exercise equipment | **One to one** with you and the therapist - **with** exercise equipment **(gym)** |
| How far you have to travel to get to the clinic | **16** miles | **2** miles |
| Parking facilities | **Ample** parking | **Limited** parking |
|  |  |  |

**Scenario 4 (IVA)**

Choose your preferred option below:

| **Characteristic** | **Option 1** | **Option 2** |
| --- | --- | --- |
| Who is treating you in the follow up treatments | **Physiotherapists** | Physiotherapy **assistants / support workers** |
| How long you have to wait to be seen after your initial physiotherapy assessment | **2** weeks | **8** weeks |
| Seeing the same or different person in your follow up treatments | Seen by **different** person | Seen by **the same** person |
| Number of follow up treatments | **8** follow up treatments | **2** follow up treatments |
| How you have your follow up treatments | In an exercise **class (gym) with** other **patients** | **One to one** with you and the therapist - **no** exercise equipment |
| How far you have to travel to get to the clinic | **4** miles | **8** miles |
| Parking facilities | **Limited** parking | **Ample** parking |
|  |  |  |

**Scenario 5 (VA)**

Choose your preferred option below:

| **Characteristic** | **Option 1** | **Option 2** |
| --- | --- | --- |
| Who is treating you in the follow up treatments | Physiotherapy **assistants / support workers** | **Physiotherapists** |
| How long you have to wait to be seen after your initial physiotherapy assessment | **8** weeks | **2** weeks |
| Seeing the same or different person in your follow up treatments | Seen by **different** person | Seen by **the same** person |
| Number of follow up treatments | **2** follow up treatments | **8** follow up treatments |
| How you have your follow up treatments | **One to one** with you and the therapist - **no** exercise equipment | **One to one** with you and the therapist - **with** exercise equipment **(gym)** |
| How far you have to travel to get to the clinic | **4** miles | **8** miles |
| Parking facilities | **Limited** parking | **Ample** parking |
|  |  |  |

**Scenario 6 VIA)**

Choose your preferred option below:

| **Characteristic** | **Option 1** | **Option 2** |
| --- | --- | --- |
| Who is treating you in the follow up treatments | Physiotherapy **assistants / support workers** | **Physiotherapists** |
| How long you have to wait to be seen after your initial physiotherapy assessment | **8** weeks | **2** weeks |
| Seeing the same or different person in your follow up treatments | Seen by **different** person | Seen by **the same** person |
| Number of follow up treatments | **8** follow up treatments | **2** follow up treatments |
| How you have your follow up treatments | In an exercise **class (gym) with** other **patients** | **One to one** with you and the therapist - **with** exercise equipment **(gym)** |
| How far you have to travel to get to the clinic | **8** miles | **4** miles |
| Parking facilities | **Ample** parking | **Limited** parking |
|  |  |  |

**Scenario 7 (VIIA)**

Choose your preferred option below:

| **Characteristic** | **Option 1** | **Option 2** |
| --- | --- | --- |
| Who is treating you in the follow up treatments | Physiotherapy **assistants / support workers** | **Physiotherapists** |
| How long you have to wait to be seen after your initial physiotherapy assessment | **4** weeks | **6** weeks |
| Seeing the same or different person in your follow up treatments | Seen by **the same** person | Seen by **different** person |
| Number of follow up treatments | **4** follow up treatments | **6** follow up treatments |
| How you have your follow up treatments | **One to one** with you and the therapist - **with** exercise equipment **(gym)** | **One to one** with you and the therapist - **no** exercise equipment |
| How far you have to travel to get to the clinic | **2** miles | **16** miles |
| Parking facilities | **Limited** parking | **Ample** parking |
|  |  |  |

**Scenario 8 (VIIIA)**

Choose your preferred option below:

| **Characteristic** | **Option 1** | **Option 2** |
| --- | --- | --- |
| Who is treating you in the follow up treatments | Physiotherapy **assistants / support workers** | **Physiotherapists** |
| How long you have to wait to be seen after your initial physiotherapy assessment | **2** weeks | **8**weeks |
| Seeing the same or different person in your follow up treatments | Seen by **the same** person | Seen by **different** person |
| Number of follow up treatments | **8** follow up treatments | **2** follow up treatments |
| How you have your follow up treatments | In an exercise **class (gym) with** other **patients** | **One to one** with you and the therapist - **no** exercise equipment |
| How far you have to travel to get to the clinic | **8** miles | **4** miles |
| Parking facilities | **Ample** parking | **Limited** parking |
|  |  |  |

**BLOCK 2**

**Scenario 1B (IB)**

Choose your preferred option below:

| **Characteristic** | **Option 1** | **Option 2** |
| --- | --- | --- |
| Who is treating you in the follow up treatments | **Physiotherapists** | Physiotherapy **assistants / support workers** |
| How long you have to wait to be seen after your initial physiotherapy assessment | **8** weeks | **2** weeks |
| Seeing the same or different person in your follow up treatments | Seen by **the same** person | Seen by **different** person |
| Number of follow up treatments | **4** follow up treatments | **6** follow up treatments |
| How you have your follow up treatments | **One to one** with you and the therapist - **with** exercise equipment **(gym)** | **One to one** with you and the therapist - **no** exercise equipment |
| How far you have to travel to get to the clinic | **8** miles | **4** miles |
| Parking facilities | **Ample** parking | **Limited** parking |
|  |  |  |

**Scenario 2B (IIB)**

Choose your preferred option below:

| **Characteristic** | **Option 1** | **Option 2** |
| --- | --- | --- |
| Who is treating you in the follow up treatments | Physiotherapy **assistants / support workers** | **Physiotherapists** |
| How long you have to wait to be seen after your initial physiotherapy assessment | **6** weeks | **4** weeks |
| Seeing the same or different person in your follow up treatments | Seen by **the same** person | Seen by **different** person |
| Number of follow up treatments | **6** follow up treatments | **4** follow up treatments |
| How you have your follow up treatments | **One to one** with you and the therapist - **no** exercise equipment | In an exercise **class (gym) with** other **patients** |
| How far you have to travel to get to the clinic | **4** miles | **8** miles |
| Parking facilities | **Ample** parking | **Limited** parking |
|  |  |  |

**Scenario 3B (IIIB)**

Choose your preferred option below:

| **Characteristic** | **Option 1** | **Option 2** |
| --- | --- | --- |
| Who is treating you in the follow up treatments | Physiotherapy **assistants / support workers** | **Physiotherapists** |
| How long you have to wait to be seen after your initial physiotherapy assessment | **2** weeks | **8** weeks |
| Seeing the same or different person in your follow up treatments | Seen by **different** person | Seen by **the same** person |
| Number of follow up treatments | **4** follow up treatments | **6** follow up treatments |
| How you have your follow up treatments | **One to one** with you and the therapist - with exercise equipment **(gym)** | In an exercise **class (gym) with** other **patients** |
| How far you have to travel to get to the clinic | **16** miles | **2** miles |
| Parking facilities | **Ample** parking | **Limited** parking |
|  |  |  |

**Scenario 4B (IVB)**

Choose your preferred option below:

| **Characteristic** | **Option 1** | **Option 2** |
| --- | --- | --- |
| Who is treating you in the follow up treatments | **Physiotherapists** | Physiotherapy **assistants / support workers** |
| How long you have to wait to be seen after your initial physiotherapy assessment | **4** weeks | **6** weeks |
| Seeing the same or different person in your follow up treatments | Seen by **different** person | Seen by **the same** person |
| Number of follow up treatments | **2** follow up treatments | **8** follow up treatments |
| How you have your follow up treatments | In an exercise **class (gym) with** other **patients** | **One to one** with you and the therapist - with exercise equipment **(gym)** |
| How far you have to travel to get to the clinic | **2** miles | **16** miles |
| Parking facilities | **Ample** parking | **Limited** parking |
|  |  |  |

**Scenario 5B (VB)**

Choose your preferred option below:

| **Characteristic** | **Option 1** | **Option 2** |
| --- | --- | --- |
| Who is treating you in the follow up treatments | **Physiotherapists** | Physiotherapy **assistants / support workers** |
| How long you have to wait to be seen after your initial physiotherapy assessment | **6** weeks | **6** weeks |
| Seeing the same or different person in your follow up treatments | Seen by **the same** person | Seen by **different** person |
| Number of follow up treatments | **6** follow up treatments | **4** follow up treatments |
| How you have your follow up treatments | **One to one** with you and the therapist - **no** exercise equipment | In an exercise **class (gym) with** other **patients** |
| How far you have to travel to get to the clinic | **2** miles | **16** miles |
| Parking facilities | **Limited** parking | **Ample** parking |
|  |  |  |

**Scenario 6B (VIB)**

Choose your preferred option below:

| **Characteristic** | **Option 1** | **Option 2** |
| --- | --- | --- |
| Who is treating you in the follow up treatments | Physiotherapy **assistants / support workers** | **Physiotherapists** |
| How long you have to wait to be seen after your initial physiotherapy assessment | **4** weeks | **6** weeks |
| Seeing the same or different person in your follow up treatments | Seen by **different** person | Seen by **the same** person |
| Number of follow up treatments | **2** follow up treatments | **8** follow up treatments |
| How you have your follow up treatments | **One to one** with you and the therapist - **with** exercise equipment **(gym)** | **One to one** with you and the therapist - **no** exercise equipment |
| How far you have to travel to get to the clinic | **8** miles | **4** miles |
| Parking facilities | **Limited** parking | **Ample** parking |
|  |  |  |

**Scenario 7B (VIIB)**

Choose your preferred option below:

| **Characteristic** | **Option 1** | **Option 2** |
| --- | --- | --- |
| Who is treating you in the follow up treatments | **Physiotherapists** | Physiotherapy **assistants / support workers** |
| How long you have to wait to be seen after your initial physiotherapy assessment | **6** weeks | **4** weeks |
| Seeing the same or different person in your follow up treatments | Seen by **different** person | Seen by **the same** person |
| Number of follow up treatments | **8** follow up treatments | **2** follow up treatments |
| How you have your follow up treatments | **One to one** with you and the therapist - **no** exercise equipment | In an exercise **class (gym) with** other **patients** |
| How far you have to travel to get to the clinic | **16** miles | **2** miles |
| Parking facilities | **Limited** parking | **Ample** parking |
|  |  |  |

**Scenario 8B (VIIIB)**

Choose your preferred option below:

| **Characteristic** | **Option 1** | **Option 2** |
| --- | --- | --- |
| Who is treating you in the follow up treatments | Physiotherapy **assistants / support workers** | **Physiotherapists** |
| How long you have to wait to be seen after your initial physiotherapy assessment | **6** weeks | **4** weeks |
| Seeing the same or different person in your follow up treatments | Seen by **the same** person | Seen by **different** person |
| Number of follow up treatments | **6** follow up treatments | **4** follow up treatments |
| How you have your follow up treatments | **One to one** with you and the therapist - **no** exercise equipment | **One to one** with you and the therapist - with exercise equipment **(gym)** |
| How far you have to travel to get to the clinic | **4** miles | **8** miles |
| Parking facilities | **Limited** parking | **Ample** parking |
|  |  |  |

**About you**

Please answer the questions about yourself which will help us check if we have collected information from a representative cross section of patients living in Staffordshire and understand better the results of the survey.

QY1: Where do you live? Please enter the first part of your postcode e.g., ST1


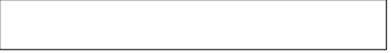


Validation requirement: maximum of 4 characters to ensure that participants do not complete their full postcode.

QY2: How would you describe your national identity? Choose all that apply

- English
- Welsh
- Scottish
- Northern Irish
- Other, write in


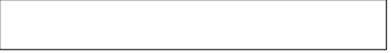


QY3: What is your ethnic group? Choose one section from A to E, then choose what best describes your ethnic group or background

**A White**

- English, Welsh, Scottish, Northern Irish or British
- Irish
- Gypsy or Irish Traveller
- Roma
- Any other White background, write in


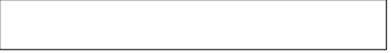


**B Mixed or Multiple ethnic groups**

- White and Black Caribbean
- White and Black African
- White and Asian
- Any other Mixed or Multiple background, write in


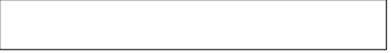


**C Asian or Asian British**

- Indian
- Pakistani
- Bangladeshi
- Chinese
- Any other Asian background, write in


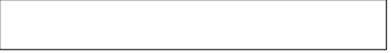


**D Black, Black British, Caribbean or African**

- Caribbean
- African background, write in


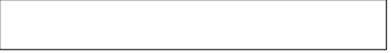


- Any other Black, Black British or Caribbean background, write in


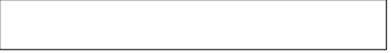


**E Other ethnic group**

- Arab
- Any other ethnic group, write in


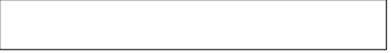


**Questions about your qualifications**

The next set of questions is about your qualifications. Record any qualifications you have ever achieved in England, Wales or worldwide, including equivalents, even if you are not using them now

**QQ1: Have you completed an apprenticeship? For example trade, advanced, foundation, modern**

- Yes
- No

**QQ2: Have you achieved a qualification at degree level or above? For example, degree, foundation degree, HND or HNC, NVQ level 4 and above, teaching or nursing**

- Yes
- No

**Q2Q: Have you achieved any other qualification? Tick all that apply**

**GCSEs or equivalent**

- 5 or more GCSEs (A* - C, 9 – 4) O levels (passes) or CSEs (grade 1)
- Any other GCSEs, O levels or CSEs (any grades) or Basic Skills course

**AS, A level or equivalent**

- 2 or more A levels
- 1 A level
- 1 AS level

**NVQ or equivalent**

- NVQ level 3
- NVQ level 2
- NVQ level 1

**Other or no qualifications**

- Any other qualifications, equivalent unknown
- No qualifications

**Questions about your employment status and household income**

The next set of questions is about your employment status and household income

**QE1: In the last 7 days, were you doing any of the following? Tick all that apply. Include casual or temporary work, even if only for one hour**

- Working as an employee
- Self-employed or freelance
- Temporarily away from work ill, on holiday or temporarily laid off
- On maternity or paternity leave
- Doing any other kind of paid work
- **OR** none of the above (Go to question 26)

**QE2: Which of the following describes what you were doing in the last seven days. Tick all that apply**

- Retired (whether receiving a pension or not)
- Studying
- Looking after home or family
- Long-term disabled
- Other

**QE3: What was your total household income before taxes during the past 12 months?**

- Less than £20,000
- £20,000 - £39,999
- £40,000 - £59,999
- £60,000 - £99,999
- More than £100,000
- Prefer not to say

**QE4**: Please use the **space below** to **enter any comments** you might want to make about this survey or **anything you might want to add** that was **not covered** in the questions above


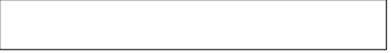


**End of survey**

We **thank you** for your time spent taking this survey. Your response has been **recorded**.

If you have any questions about the survey or if you would like any further information,

please contact Panos Sarigiovannis **via email**: [p.sarigiovannis1@keele.ac.uk](mailto:p.sarigiovannis1@keele.ac.uk)


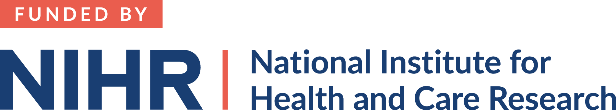

Supplement: Supplementary file 1 — Supplementary Material 1 [file 12913_2024_11585_MOESM1_ESM.docx]
